# Strong exciton-photon coupling in self-hybridized organic–inorganic lead halide perovskite microcavities

**DOI:** 10.1515/nanoph-2023-0366

**Published:** 2023-11-08

**Authors:** Zeeshan Tahir, Jin-Woo Jung, Mamoon Ur Rashid, Sungdo Kim, Dinh Khoi Dang, Jang-Won Kang, Chang-Hee Cho, Joon I. Jang, Yong Soo Kim

**Affiliations:** Department of Semiconductor Physics and Energy Harvest Storage Research Center, University of Ulsan, Ulsan 44610, South Korea; Faculty of Chemical and Food Technology, Ho Chi Minh City University of Technology and Education, Ho Chi Minh City, Vietnam; Department of Physics and Chemistry, Daegu Gyeongbuk Institute of Science & Technology (DGIST), Daegu 42988, South Korea; Department of Semiconductor and Applied Physics, Mokpo National University, Muan 58554, South Korea; Department of Physics, Sogang University, Seoul 04017, South Korea

**Keywords:** strong coupling, exciton-polaritons, self-hybridized optical microcavities, organic-inorganic lead halide perovskites

## Abstract

Controlling coherent light–matter interactions in semiconductor microcavities is at the heart of the next-generation solid-state polaritonic devices. Organic–inorganic hybrid perovskites are potential materials for room-temperature polaritonics owing to their high exciton oscillator strengths and large exciton binding energies. Herein, we report on strong exciton-photon coupling in the micro-platelet and micro-ribbon shaped methylammonium lead bromide single crystals. Owing to high crystallinity and large refractive index, the as-grown perovskite microcrystals serve as self-hybridized optical microcavities along different orientations due to their distinct physical dimensionalities. In this regard, the perovskite micro-platelet forms a simple Fabry–Perot microcavity in out-of-plane orientation, while the micro-ribbon functions as a Fabry–Perot type waveguide microcavity within the plane of the perovskite sample. Consequently, excitons in these microcavities strongly interact with their corresponding uncoupled cavity modes, yielding multimode exciton-polaritons with Rabi splitting energies ∼205 and 235 meV for micro-platelet and micro-ribbon geometry, respectively. Furthermore, micro-ribbon geometry displays Young’s double-slit-like interference patterns, which together with the numerical simulation readily reveals the parity and the mode order of the uncoupled cavity modes. Thus, our results not only shed light on strong exciton-photon coupling in various morphologies of methylammonium lead bromide microcrystals but also open an avenue for advanced polaritonic devices.

## 1 Introduction

Exciton-polaritons are Bosonic quasiparticles formed due to strong coupling between excitons (electron-hole pairs) and cavity photons in a semiconductor microcavity [1]. In order to realize strong coupling, the energy exchange rate between excitons and cavity photons should be faster than their individual dissipation rates [2]. Interestingly, the resultant solid-state polaritons exhibit both exciton-like and photon-like characteristics such as the low effective mass (typically 10^−4^ times the electron mass), fast propagation, and high spatial coherence inherited from the photonic constituent while the strong nonlinearities acquired from the excitonic counterpart [3, 4]. These superior features not only make exciton–polaritons an ideal platform for studying super fascinating quantum optical phenomena including Bose–Einstein condensation [5, 6], superfluidity [7], quantum vortices [8], optical spin Hall effect [9], but are also responsible for numerous advanced optoelectronic applications such as inversionless polariton lasers [10, 11], neuromorphic computing [12], polariton optical switches [13], and slow light devices [14].

Experimentally, strong exciton–photon coupling (*g*) in solid-state systems demands a large exciton oscillator strength (*f*), a high spatial overlap between the excitonic medium and photonic field (*N*) and a low cavity mode volume (*v*_*m*_) as per the expression, $g\propto \sqrt{Nf/v_{m}}$ [15, 16]. Pertinently, a plethora of inorganic materials have been widely investigated ranging from narrow-bandgap semiconductors (GaAs, InAs, and InP) [17–19] with cryogenic-limited excitons to wide-bandgap semiconductors (ZnO, GaN) [20, 21] with robust excitons at room temperature. Recently, transition metal dichalcogenide quantum wells encapsulated in planar microcavities have shown immense potential towards low-threshold polariton lasing and condensation thanks to their enhanced *f* and large exciton binding energies (typically a few hundred meV) [22–24]. However, engineering planar microcavities not only requires sophisticated epitaxial techniques that usually suffer built-in strain resulting from thermal expansion mismatch but also offers a limited spatial overlap due to the small thickness of the active (excitonic) medium [25]. In contrast, organic semiconductors owing to their large exciton binding energies, ease of fabrication and the ability to form self-assembled optical microcavities provide an alternative platform for room-temperature polaritonics [26, 27]. Nonetheless, the tightly bound Frenkel exciton in these organic microcavities typically exhibit weak Coulombic interactions, resulting in higher thresholds and weaker nonlinearities [14]. Therefore, the optimal solution is to develop hybrid organic-inorganic semiconductors that can essentially unify the beneficial characteristics of both constituents [16, 28, 29].

Organic-inorganic hybrid perovskites (OIHPs), owing to their extraordinary photophysical properties such as high quantum efficiencies [30], large exciton binding energies [31–33], broad bandgap tunability [34], long carrier diffusion lengths [35], and low trap densities [36], have drawn enormous attraction for application in light-emitting diodes [37], photodetectors [38], solar cells [39], and micro-lasers etc. [40]. Particularly, OIHP microcrystals are ideal for realizing miniature lasers with considerably low thresholds owing to their facile synthesis and the capability to simultaneously act as a gain medium and an optical cavity offering a high spatial overlap [41]. Moreover, the emission orientation in OIHP microcrystals can be easily tuned via altering the crystal geometry, yielding various types of optical microcavities such as simple Fabry–Perot (FP) cavities [42], FP-type waveguides [6, 43], coupled waveguides [44], and whispering gallery modes [45]. Based on the cumulative effect of these characteristics, OIHP microcrystals are exciting avenues for studying strong exciton–photon interactions in epitaxy-free microcavities [46–48].

In this work, we have investigated strong exciton–photon coupling in micro-platelet (MP) and micro-ribbon (MR) shaped methylammonium lead bromide (CH_3_NH_3_PbBr_3_) single crystals grown via the spaced-confined anti-solvent crystallization method. The as-grown CH_3_NH_3_PbBr_3_ microcrystals exhibit sharp and featureless facets, high crystallinity, and large refractive index contrast with respect to the surrounding. Consequently, these perovskite microcrystals form self-assembled optical microcavities, however along different orientations owing to their distinct geometries. For instance, MP geometry forms a simple FP microcavity in the out-of-plane direction, while MR serves as an FP-type waveguide microcavity within the plane of the perovskite sample. Moreover, the large oscillator strength and high binding energy (*E*_*B*_ = 50–60 meV) [32, 33] of the exciton also signifies the potential possibility of room-temperature strong exciton–photon coupling in CH_3_NH_3_PbBr_3_ perovskite microcrystals. Interestingly, the angle-resolved photoluminescence (ARPL) mappings show multiple lower polariton branches (LPBs), whereby a successive coupled oscillator model (COM) fitting reveals the Rabi splitting energies (*ħΩ*) ∼205 and 235 meV for MP and MR geometries, respectively. Besides, the Young’s double-slit-like interference patterns of the LPBs in the MR geometry straightforwardly reveal the parity and mode order of the associated uncoupled cavity modes, which is consistent with the spatial electric field distribution obtained by a finite difference time domain (FDTD) simulation. Thus, our results not only explore the strong exciton-photon coupling in self-hybridized CH_3_NH_3_PbBr_3_ microcavities but also highlight their potential towards the realization of practical polaritonic devices working at room temperature.

## 2 Results and discussion

**Figure 1:**
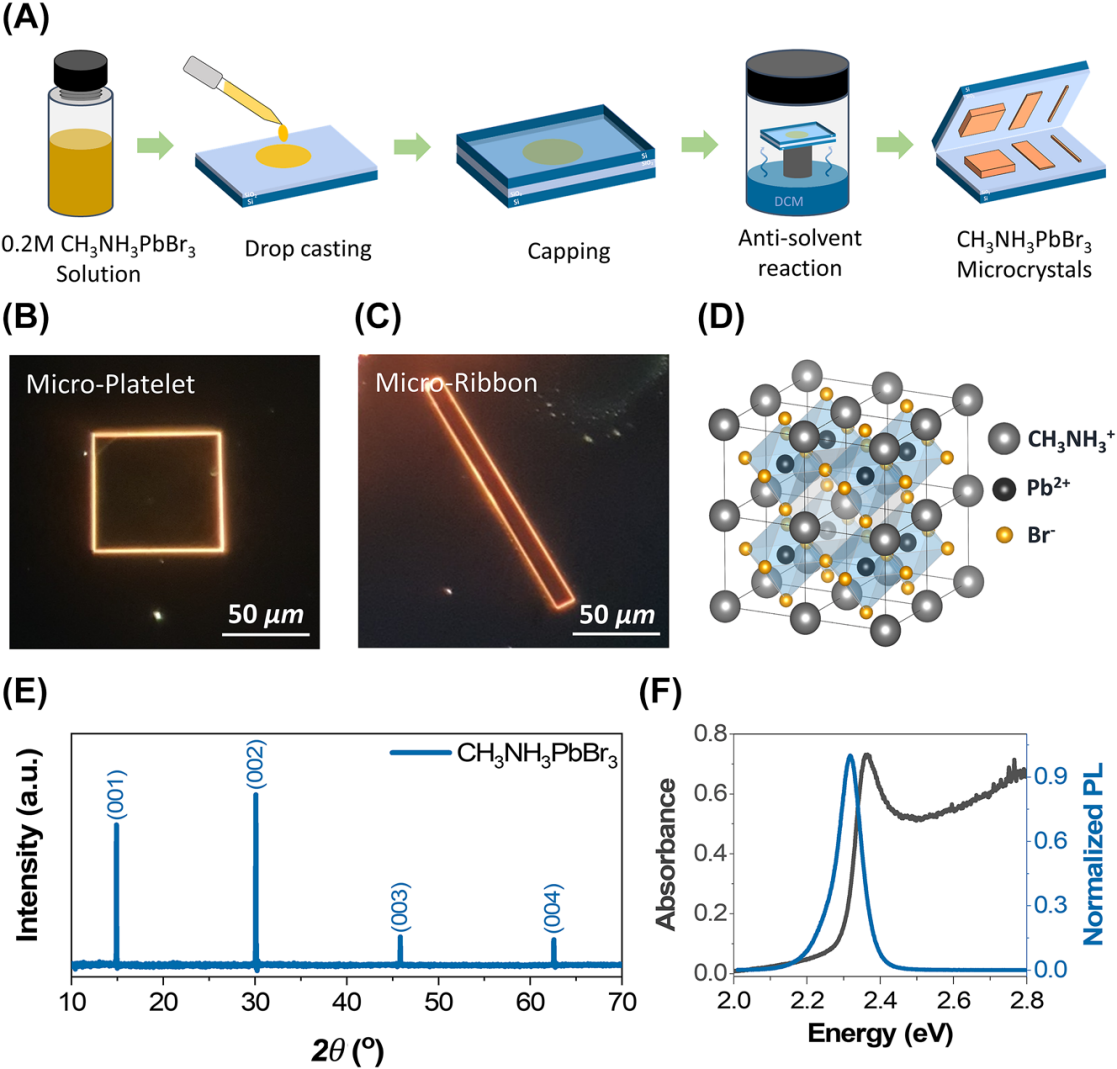
Growth and characterization of CH_3_NH_3_PbBr_3_ microcrystals. (A) The systematic growth process of the CH_3_NH_3_PbBr_3_ microcrystals. (B and C) The dark-field OM images of the MP and MR shaped CH_3_NH_3_PbBr_3_ microcrystals, respectively. The images reveal sharp and well-defined boundaries/edges of the microcrystals signifying their potential as self-assembled optical microcavities. (D) Schematic of the cubic crystal structure of CH_3_NH_3_PbBr_3_. (E) The XRD pattern of CH_3_NH_3_PbBr_3_. The pattern reveals the single crystalline nature and the cubic phase of the as-grown CH_3_NH_3_PbBr_3_ microcrystals. (F) The room-temperature absorbance and PL spectra of CH_3_NH_3_PbBr_3_. The absorbance spectrum (black) shows a clear and strong excitonic peak at 2.365 eV, while the PL spectrum displays strong excitonic emission at 2.317 eV.

CH_3_NH_3_PbBr_3_ microcrystals with different morphological forms were grown via the spaced-confined anti-solvent crystallization technique illustrated systematically in Figure 1A (details in the experimental section). Figure 1B and C present the dark-field optical microscope (OM) images of MP and MR shaped CH_3_NH_3_PbBr_3_ crystals, respectively. The images reveal sharp and well-defined facets/edges signifying their potential to function as self-hybridized optical microcavities. The crystalline quality and phase/structure of the as-grown CH_3_NH_3_PbBr_3_ microcrystals was investigated by X-ray diffraction (XRD). Previous studies suggest that the thermodynamically stable phase of CH_3_NH_3_PbBr_3_ at room temperature is cubic, comprised of corner-sharing lead bromide octahedra surrounded by CH_3_NH_3_ cations as shown in Figure 1D. Figure 1E shows the XRD pattern of the as-grown CH_3_NH_3_PbBr_3_ microcrystals, wherein the sole existence of the (00h) series diffraction peaks not only reflects the single crystalline nature but also confirms the cubic crystal structure of the our perovskite microcrystals [32]. Moreover, the realization of exciton–polaritons at room temperature demands an exciton binding energy (*E*_*B*_) > 25 meV making them robust against thermal fluctuation at room temperature. Literature suggest that CH_3_NH_3_PbBr_3_ exhibits stable excitons with *E*_*B*_ ∼50–60 meV [32, 33], which is also confirmed by our room-temperature absorption spectrum (Figure 1F: black curve), showing a clear excitonic peak centered at ∼2.365 eV. Likewise, the room-temperature photoluminescence (PL) spectrum (Figure 1F: blue trace) demonstrates strong and efficient emission as indicated by the sharp peak at ∼2.317 eV with a narrow full-width half maximum (FWHM) ∼74 meV, which is in good agreement with previous reports [33, 49].

**Figure 2:**
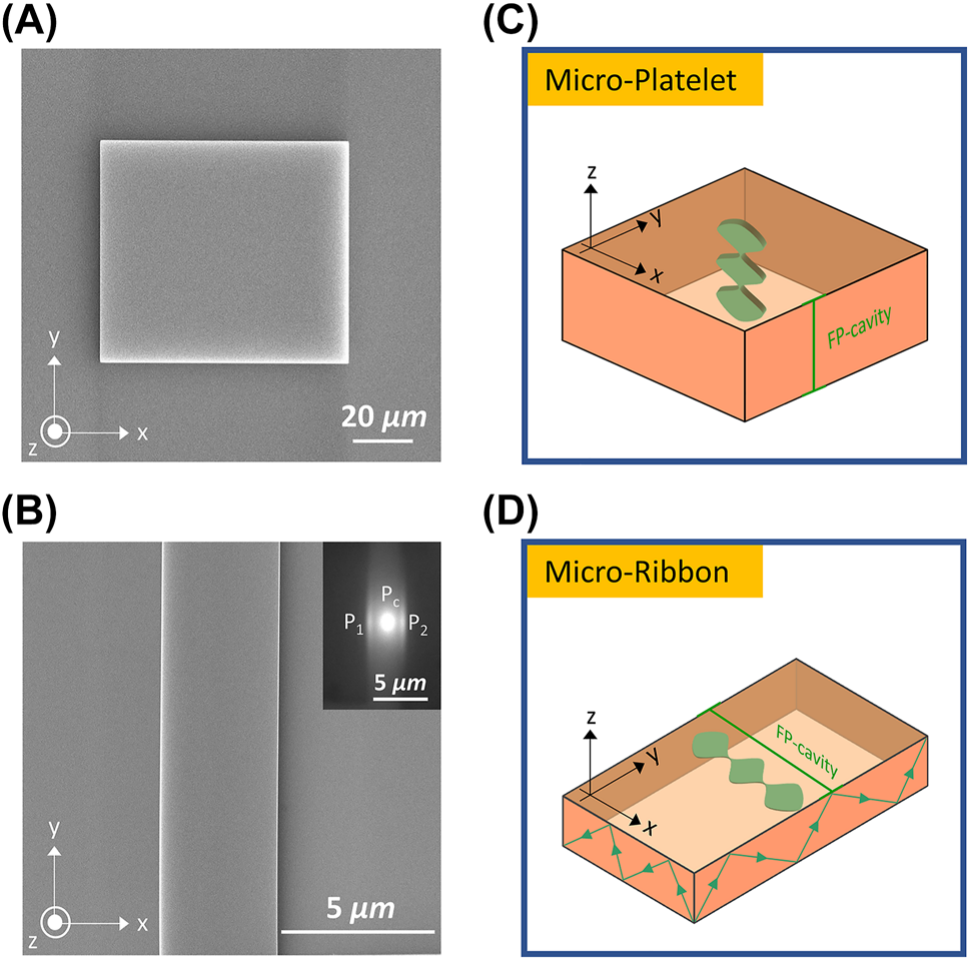
CH_3_NH_3_PbBr_3_ microcrystals as self-assembled optical microcavities. (A, B) SEM images of MP and MR, respectively. The images show uniform and featureless surfaces with corresponding lateral (*x*–*y*) dimensions ∼80 × 70 and 15 × 3.87 µm^2^. (C, D) Schematic representations of MP and MR geometries, indicating the type and orientation of the self-hybridized optical microcavities. As evident, MP forms a simple FP along the *z*-orientation, while MR serves as an FP-type waveguide microcavity along the *x*-axis.

Figure 2A and B present SEM images of the perovskite MP and MR, respectively. The images show uniform and featureless surfaces with corresponding lateral (*x*–*y*) dimensions ∼80 × 70 and 15 × 3.87 µm^2^. Their representative thicknesses (*z*-dimension) or height profiles measured by laser scanning confocal microscope are ∼3.67 and 0.378 µm (Figure S2). Benefited from the high crystallinity, sharp facets (evident from dark-field OM and SEM images) and large refractive index contrast relative to the surrounding [50], both perovskite geometries form self-hybridized optical microcavities along different orientations due to their distinct physical dimensions. For instance, the perovskite MP forms a simple FP cavity in the out-of-plane (*z*-axis) orientation as shown schematically in Figure 2C [14]. While MR forms an FP-type waveguide microcavity along the shorter *x*-axis, wherein the guided modes propagate along the lateral (*x*–*y*) dimension via total internal reflection in a zig-zag fashion as illustrated in Figure 2D. The grayscale PL image (inset of Figure 2B) readily confirms the formation of the FP-type waveguide cavity owing to the emission from the edges (*P*_1_ and *P*_2_) of MR upon excitation at the center (*P*_*c*_), signifying the waveguiding behavior, consistent with the previous reports [6, 43].

**Figure 3:**
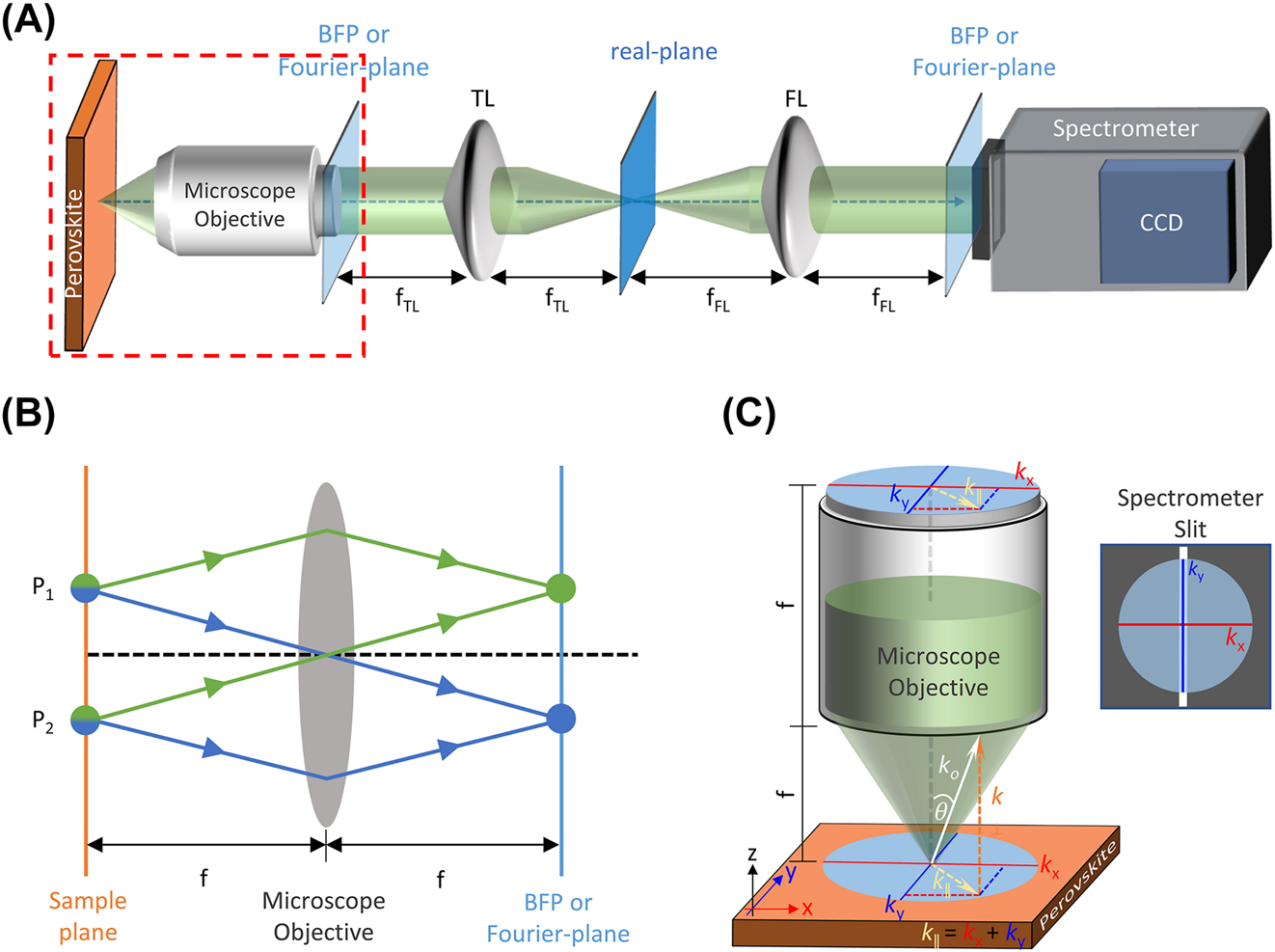
ARPL spectroscopy. (A) Schematic of the ARPL setup depicting the projection of the BFP on the spectrometer slit. (B) The ray diagram illustrates that the BFP of the microscope objective holds the angle information of the sample kept at its front focal point. (C) Enlarged view of the highlighted (red) region in Figure 3A. The image shows that the BFP is basically the in-plane component (*k*_‖_ = *k*_*x*_ + *k*_*y*_) of the wavevector (*k*_0_) collected by the microscope objective at an angle (*θ*). The right panel displays a schematic of the BFP projection on the spectrometer slit.

Conventionally, strong exciton–photon coupling in a semiconductor microcavity system is probed via the ARPL, also known as the back focal plane (BFP)/Fourier plane or *k*-plane spectroscopy. Figure 3A presents the schematic of an ARPL spectroscopy setup, which consists of a microscope objective, a tube lens (TL), a Fourier lens (FL) and a spectrometer arranged in a 4*f*-relay configuration with the aim to project the BFP of the microscope objective on the spectrometer entrance slit. This is because the BFP encrypts the angular information of the test sample as demonstrated simply in the ray diagram (Figure 3B), where the light collected by the microscope objective from two different points (*P*_1_ and *P*_2_) in the sample plane with the same angle (color coded: green and blue) tends to converge at a single point on the BFP, thereby manifesting that each point on BFP corresponds to the light collected by objective lens at a certain unique angle. Further, the correlation between BFP and angle (*θ*) in terms of the electromagnetic wavevector (*k*) is presented graphically in Figure 3C. The image displays a schematic of the light cone collected by the microscope objective (enlarged view of the highlighted region in Figure 3A), wherein the resolution of wavevector (*k*_0_) into in-plane (*k*_‖_ = *k*_0_ sin *θ*) and out-of-plane (*k*_⊥_ = *k*_0_ cos *θ*) components reveals that the BFP of the microscope objective is nothing but the projection of *k*_‖_ = *k*_*x*_ + *k*_*y*_ components of the wavevectors collected at various angles. Thus, changing the orientation of the sample with respect to the microscope stage in ARPL spectroscopy would in return change the orientation of the BFP relative to the spectrometer slit, thereby offering the freedom to selectively resolve the in-plane *k*_*x*_ and *k*_*y*_ components as shown schematically in the left panel of Figure 3C.

**Figure 4:**
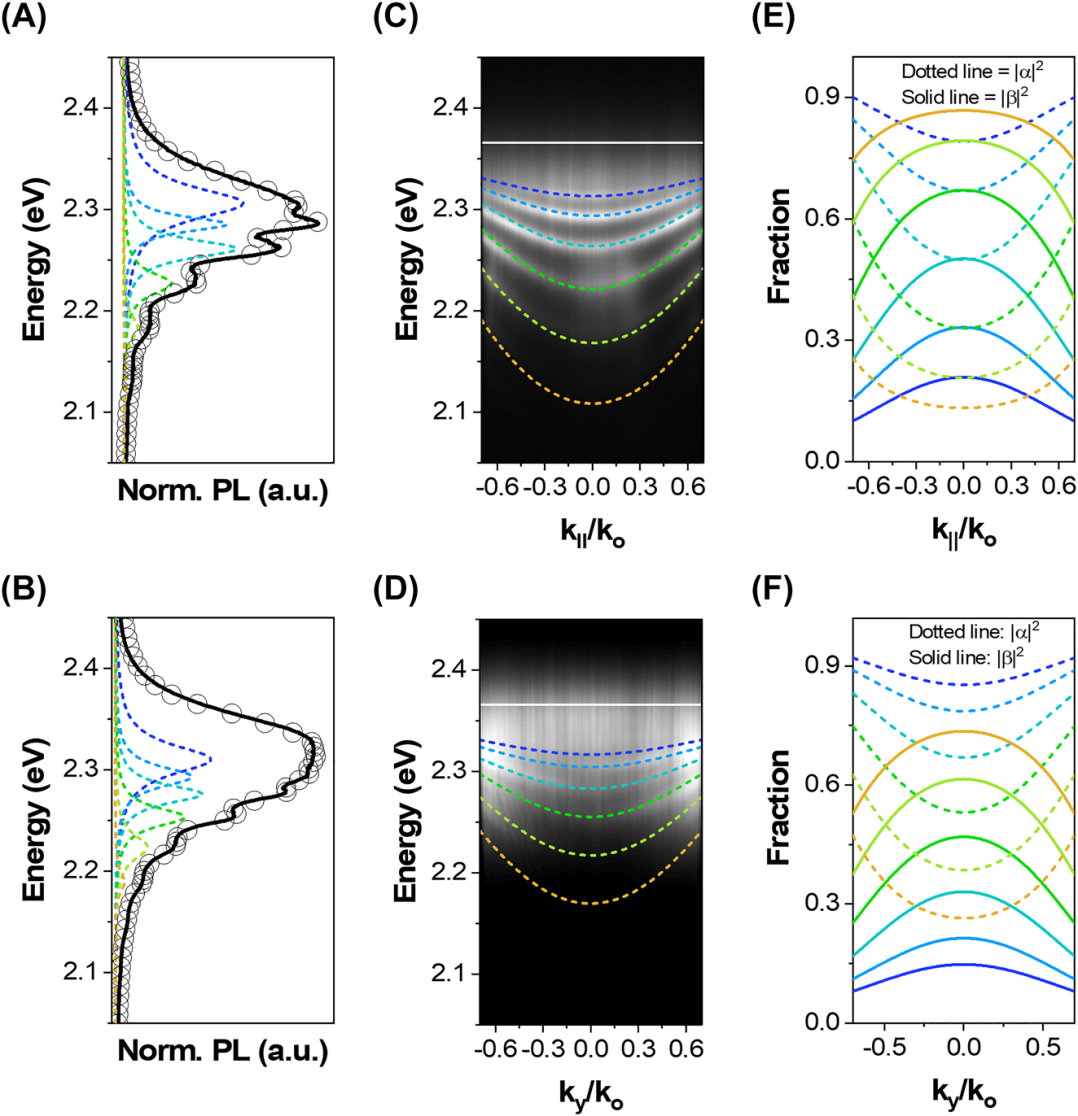
Strong exciton–photon coupling in perovskite MP and MR geometries. (A, B) The steady-state PL spectra of MP and MR, respectively. The spectra show multiple resonances with unequal adjacent spacing, all well fitted by multi-Lorentzian functions, where the circles represent their respective/corresponding cumulative fits. The presence of these multiple resonance not only confirms the formation of self-hybridized optical microcavities, but also signifies the strong exciton–photon coupling owing to the adjacent asymmetric resonance spacing. The ARPL mappings of (C) MP and (D) MR, when the *y*-axis of MR is set parallel to the spectrometer slit. The mappings present a clear parabolic dispersion for each of the modes observed in the steady-state PL spectra. The theoretical COM fitting (dotted lines) confirms that these parabolic dispersions are indeed the lower polariton branches formed due to strong coupling between the excitons (solid white line) and multiple uncoupled cavity modes of MP and MR geometries. (E, F) Hopfield coefficients indicating the excitonic |*α*|^2^ and photonic |*β*|^2^ fractions of the individual polariton modes observed in MP and MR geometries, respectively.

Figure 4A and B show the steady-state PL spectra of the perovskite MP and MR, respectively. As expected, multiple resonances were observed in the corresponding PL spectra, which not only confirm the formation of self-hybridized optical cavities but also suggests strong exciton–photon coupling owing to the asymmetric spacing between the adjacent resonances (details in Table S1). To ensure the strong exciton–photon coupling, ARPL spectroscopy was conducted. Figure 4C and D present the ARPL mappings of MP and MR, respectively. The mappings present clear parabolic dispersions for each of the modes/resonances observed in the corresponding PL spectra (Figure 4A and B). Additionally, obvious signs of strong-exciton photon coupling were observed in the ARPL mappings such as the flattening of dispersion curves and decrease in the energy spacing of the dispersion curves as the mode energy approaches the exciton level (white solid line). This behavior is well consistent with the previous reports on strong exciton–photon coupling in self-assembled semiconductor microcavities [15, 47]. Figure S3 present the corresponding angle resolved reflectance (ARR) mappings of MP and MR while Figure S4 displays a comparison between the experimental and the transfer matrix method simulated ARR mapping of MP geometry (details in Notes S1 and S2, respectively). Further, to get a deeper insight into the strong exciton–photon coupling and estimate the characteristic *ħ*Ω, the experimentally measured ARPL mappings were fitted (dotted line) via the theoretical COM [15]

(1) $$\left[\begin{array}{cc} E_{X} & g\\ g & E_{CM} \end{array}\right]\left[\begin{array}{c} \alpha \\ \beta \end{array}\right]=E_{\text{UPB/LPB}}\left[\begin{array}{c} \alpha \\ \beta \end{array}\right]$$

where *E*_*X*_ is the exciton resonance energy (obtained from the absorption spectrum), *g* is the exciton–photon coupling strength (fitting parameter), *E*_UPB∕LPB_ indicates the upper/lower polariton energy, while *E*_*CM*_ corresponds to the uncoupled cavity mode. Details regarding the calculation of *E*_*CM*_ for MP and MR geometry are explained in Note S3. From Equation (1), the energy of upper polariton branch (UPB) and lower polariton branch (LPB) can be approximated as

(2) $$E_{\text{UPB/LPB}}=\frac{1}{2}\left(E_{CM}+E_{X}\right)\pm \frac{1}{2}\sqrt{{\left(2g\right)}^{2}+{\left(E_{X}-E_{CM}\right)}^{2}}$$

Solving Equation (2) systematically for *E*_*CM*_ shown in Figure S5(A) and (B), ARPL mappings of Figure 4C and D were best fitted for *g* = 0.1025 eV and 0.1175 eV, respectively. The associated *ħ*Ω values estimated via the equation *ħ*Ω = 2 *g* are ∼205 and 235 meV. Thus, COM fitting verifies that the multiple parabolic dispersions observed in the ARPL mappings are indeed the LPBs formed due to strong coupling between excitons and the uncoupled *E*_*CM*_ of MP and MR geometries plotted in Figure S5(A) and (B), respectively. These *ħ*Ω values obtained from the COM fitting are in good agreement with *ħ*Ω values acquired from the *E–k* plots i.e., ∼203 and 238 meV for MP and MR as shown in Figure S6(A) and (B) **,** respectively, (details in Note S4) suggesting that both COM fitting and *E*–*k* dispersions are self-consistent. Fundamentally, the coherent energy exchange between the excitonic and the photonic states in a strongly coupled system yields both UPBs and LPBs. However, in case of MP and MR, UPBs are severely damped and can hardly be identified in the ARPL mappings because of the rapid and nonradiative depopulation of excited UPBs into the exciton reservoir, which is the valid polariton relaxation mechanism typically observed in microcavities with large Rabi-splitting [16, 25, 51].

Furthermore, the excitonic (|*α*|^2^) and photonic (|*β*|^2^) fractions of the individual polariton branches were approximated via the Hopefield coefficients as ${\left|\alpha \right|}^{2}=\frac{1}{2}\left(1+\frac{\Delta E}{\sqrt{\Delta E^{2}+4g^{2}}}\right)$ and ${\left|\beta \right|}^{2}=\frac{1}{2}\left(1-\frac{\Delta E}{\sqrt{\Delta E^{2}+4g^{2}}}\right)$ where Δ*E* = *E*_*CM*_ − *E*_*X*_ at *k*_||_/*k*_*y*_ = 0 and $\left|\alpha \right|$ ^2^ + $\left|\beta \right|$ ^2^ = 1. Figure 4E and F present the Hopfield coefficients of MP and MR geometries, where dotted and solid lines represent $\left|\alpha \right|$ ^2^ and $\left|\beta \right|$ ^2^, respectively. As evident, $\left|\alpha \right|$ ^2^ increases while $\left|\beta \right|$ ^2^ decreases as the energy of the polariton mode approaches the exciton resonance and vice versa. For example, the polariton mode (dotted: dark blue) adjacent to exciton dispersion (solid: white) demonstrates the highest $\left|\alpha \right|$ ^2^ while the one (dotted: orange) away from exciton dispersion manifests maximal $\left|\beta \right|$ ^2^, implying that MP and MR exhibit polaritons with different excitonic and photonic contents at room temperature, thereby providing an ideal testbed for studying the many body physics [52].

Note that Figure 4D is acquired when the *y*-axis of MR is parallel to the spectrometer slit as illustrated in Figure S7(A). This is because angular information in ARPL spectroscopy is directly correlated to the in-plane wavevector (*k*_‖_ = *k*_*x*_ + *k*_*y*_) as explained in Figure 3 and unlike MP geometry, where both *k*_*x*_ and *k*_*y*_ are free due to the long lateral dimensions (several 10 μm). The *k*_*x*_ of MR is confined due to its small width (*x*-axis), while the *k*_*y*_ is free owing to the long length (*y*-axis). Therefore, the ARPL mappings of MR highly depend on its orientation with respect to the spectrometer entrance slit.

**Figure 5:**
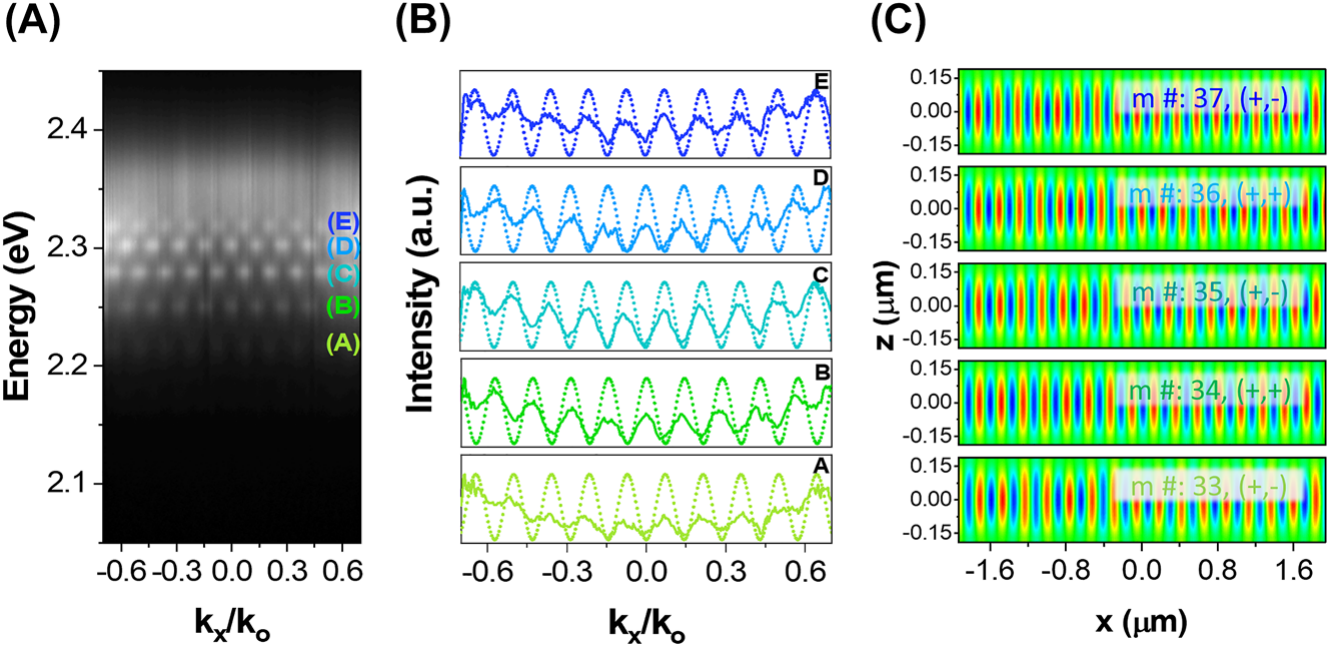
Young’s double-slit-like interference patterns of MR geometry. (A) The ARPL mapping when the short axis (*x*-axis) of MR is set parallel to the spectrometer entrance slit. The mapping presents Young’s double-slit-like interference patterns corresponding to LPBs with energy positions identical to Figure 4D at *k*_*y*_/*k*_0_ = 0. (B) The experimental (solid) and the simulated (dotted) interference patterns extracted from Figure 5A. (C) The FDTD simulated spatial electric field distribution of the LPBs in the *x*–*z* plane of the perovskite MR, where the mode numbers associated with the uncoupled cavity modes were estimated via simply counting the electric field patterns.

In order to study the phase correlation between the emissions from the edges (end emissions), the *x*-axis of MR is aligned to the spectrometer entrance slit forming a Young’s double-slit-like experimental configuration as shown schematically in Figure S7(B). Consequently, multiple standing waves like interference patterns were observed in the ARPL mapping (Figure 5A) resulting from the relative phase difference between the two optical fields (end emissions) as defined by the width of MR [43]. Each of these interference patterns represents characteristic LPB with their energy positions identical to ones shown in Figure 4D at *k*_*y*_/*k*_0_ = 0. Interestingly, these interference patterns straightforwardly reveal the parity of the associated *E*_*CM*_. Figure 5B shows the interference patterns (solid curves) extracted from the corresponding ARPL mapping (Figure 5A **)**. Clearly, the interference patterns labelled as A, C, and E show a dip, while B and D present a peak at *k*_*x*_/*k*_0_ = 0, signifying the destructive and constructive interference, respectively. This behavior can easily be interpreted by taking the parity of the cavity modes into consideration as explained in Note S5, which states that the phase difference between two point sources will be an odd multiple of *π* for odd modes (+, −) and an even multiple of *π* for even modes (+, +). Following Equations (S4) and (S5), the experimentally measured interference patterns were reproduced theoretically (dotted curves), thereby confirming that A, C, and E indeed correspond to the odd cavity modes, while B and D are associated with the even cavity modes. Finally, the spatial electric field (*E*-field) distribution and the exact mode order of *E*_*CM*_ responsible for exciton–polariton formation in the perovskite MR were computed via the FDTD simulations. Figure 5C presents the spatial *E*-field distribution of *E*_*CM*_ in the *x–z* plane of MR geometry, whereby the mode orders were extracted by counting the number of *E*-field distribution patterns. Accordingly, the mode number corresponding to the interference patterns *A*, *B*, *C*, *D*, and *E* are 33, 34, 35, 36, and 37, respectively. Intriguingly, the spatial *E*-field distribution of the odd (even) modes exhibits asymmetric (symmetric) amplitudes at the edges, manifesting a phase difference of odd (even) multiple of *π*, which is not only consistent with Figure 5B but is also in accordance with the previous reports on MR geometry [5, 42]. Thus, the ARPL mapping of in-plane microcavities together with FDTD simulations is excellent probes for estimating the parity and order of the cavity modes. Finally, the relationship between Rabi-splitting (*ħΩ*) and cavity quality factor (*Q*) is illustrated by performing the ARPL and corresponding steady-state PL spectroscopy of multiple MPs and MRs geometries as shown in Figures S8 and S9, respectively (details in Note S6). The *ħΩ* versus *Q* plots (Figure S10) describe the relation as a direct one i.e., *ħΩ* increases with increase in *Q* and vice versa for both MP and MR geometries.

## 3 Conclusions

In conclusion, we have demonstrated strong exciton–photon coupling in the MP and MR shaped CH_3_NH_3_PbBr_3_ single crystals grown via the space-confined anti-solvent crystallization method. Thanks to the high crystalline quality and the large index contrast with respect to the surrounding, the as-grown CH_3_NH_3_PbBr_3_ microcrystals function as self-hybridized optical microcavities. For instance, the perovskite MP forms a simple FP cavity in the out-of-plane direction, while MR forms an FP-type waveguide microcavity within the plane of the sample. In addition, CH_3_NH_3_PbBr_3_ also exhibits robust excitons and large oscillator strength at room temperature, thereby fulfilling all the essential prerequisites for room-temperature strong exciton-photon coupling. Consequently, multiple LPBs were successfully observed in the ARPL mappings resulting from the strong coupling between excitons and uncoupled cavity modes of MP and MR, wherein the associated *ħΩ* values were approximated via COM fitting as ∼205 and 235 meV. Besides, the parity and mode order of the uncoupled cavity modes responsible for the multimode exciton–polariton formation in MR geometry were obtained directly from the interference patterns of the LPBs and the associated FDTD simulated spatial *E*-field distribution patterns. Thus, our work offers a simple method to design epitaxy-free microcavity systems with various geometries for studying the strong light–matter interactions at room temperature.

## 4 Experimental section

### 4.1 Materials

All the chemicals used for the growth of CH_3_NH_3_PbBr_3_ microcrystals such as CH_3_NH_3_Br (methylammonium bromide 99 %), PbBr_2_ (lead bromide 99 %), DMF (Dimethylformamide 99 %) and DCM (Dichloromethane 99 %) were purchased from Sigma Aldrich. The silicon substrates coated with 300-nm-thick silicon oxide (SiO_2_) were purchased from ITASO.

### 4.2 Growth of the perovskite microcrystals

Firstly, a 0.2 M CH_3_NH_3_PbBr_3_ solution was prepared by dissolving 22 mg of CH_3_NH_3_Br and 73 mg of PbBr_2_ in 1 mL of DMF. The solution was kept at 60 °C for 6 h until it turned transparent. Afterward, 5 µL of the as-prepared CH_3_NH_3_PbBr_3_ solution was dropped on SiO_2_/Si substrate followed by an immediate capping with another substrate. The substrates were then placed in a tightly sealed Teflon vial containing 10 mL of DCM. After 24 h, CH_3_NH_3_PbBr_3_ microcrystals with various shapes/geometries and sizes were obtained on both sides of the substrates as shown in Figure S1.

### 4.3 Characterizations

The room temperature steady-state PL spectra were acquired using a 473 nm continuous-wave laser focused onto the sample surface via a 50*x*/0.5 (magnification/numerical aperture) microscope objective while the ARPL mappings were obtained via a 40*x*/0.7 objective lens covering an angular range of ±44.4°. The absorbance spectra of the CH_3_NH_3_PbBr_3_ microcrystals were measured using a homebuilt micro-UV-Vis spectrometer. The XRD 2 *θ* scan was performed via a lab source (4-circle) Bruker, D8 Discover X-ray diffractometer using Cu K_α_ as the X-ray source. The dark-field OM images were captured by a Nikon OPTIPHOT-100 microscope, while the scanning electron microscope (SEM) images were obtained by an SNE-4500M microscope. The height (thickness) profiles were measured by a VK-X200, Keyence 3D laser scanning confocal microscope. While the spatial distribution of the electric field along the *x–z* of MR geometry was simulated by the FDTD (Lumerical solutions, Inc.) implying the perfectly matching layers (PML) as the boundary condition to solve the maxwell equations. In the model, the dimensions of MR were 15 × 3.87 × 0.378 µm^3^ while the mesh size was set to ∼1 nm. The transfer matrix method (TMM) simulations were performed via MATLAB.

## Supporting Information

Bright-field optical microscope image of CH_3_NH_3_PbBr_3_ microcrystals with different morphologies, height profiles, peak positions and corresponding FWHM of the multiple resonances observed in the PL spectra of the perovskite geometries, angle-resolved reflectance (ARR) and ARPL mappings, ARR spectroscopy of MP and MR, comparison of the experimental and the TMM simulated ARR mappings of MP geometry, experimental versus TMM simulated ARR mapping of perovskite MP, calculation of uncoupled cavity modes, dispersion of LPBs and their associated uncoupled cavity modes, *E*–*k* plots, *E*–*k* dispersion calculation, schematic representation of MR orientation with respect to the spectrometer slit, equations for even and odd modes in Young’s double slit experiment, Rabi-splitting versus cavity quality factor, ARPL mappings and steady-state PL spectra of MPs with varying thicknesses, ARPL mappings and steady-state PL spectra of MRs with varying widths and *ħΩ* versus *Q* plots.

## Supplementary Material

Supplementary Material Details
