# Supplementary material for: Strong exciton-photon coupling in self-hybridized organic–inorganic lead halide perovskite microcavities
Source: Nanophotonics. 2023 Nov 8;12(23):4297–306. doi: 10.1515/nanoph-2023-0366 (PMC11501772; doi:10.1515/nanoph-2023-0366)
Supplement: Supplementary file 1 — Supplementary Material Details [file j_nanoph-2023-0366_suppl_001.docx]

**Supplementary Information**

**Strong Exciton-Photon Coupling in Self-Hybridized Organic-Inorganic Lead Halide Perovskite Microcavities**

*Zeeshan Tahir, Jin-Woo Jung, Mamoon Ur Rashid, Sungdo Kim, Dinh Khoi Dang, Jang-Won Kang*, Chang-Hee Cho*, Joon I. Jang* and Yong Soo Kim**

Zeeshan Tahir and Jin-Woo Jung have contributed equally to this work.

***Corresponding authors: Jang-Won Kang,** Department of Semiconductor and Applied Physics, Mokpo National University, Muan 58554, South Korea, E-mail: kangjw@mokpo.ac.kr (J. W. Kang); **Chang-Hee Cho,** Department of Physics and Chemistry, Daegu Gyeongbuk Institute of Science & Technology (DGIST), Daegu 42988, South Korea, E-mail: chcho@dgist.ac.kr (C. H. Cho); **Joon I. Jang,** Department of Physics, Sogang University, Seoul 04017, South Korea,

E-mail: jjcoupling@sogang.ac.kr (J. I. Jang); and **Yong Soo Kim,** Department of Semiconductor Physics and Energy Harvest Storage Research Center, University of Ulsan, Ulsan 44610, South Korea, Email: yskim2@ulsan.ac.kr (Y.S. Kim)

**Zeeshan Tahir, Mamoon Ur Rashid, Sungdo Kim, Dinh Khoi Dang,** Department of Semiconductor Physics and Energy Harvest Storage Research Center, University of Ulsan, Ulsan 44610, South Korea

**Dinh Khoi Dang,** Faculty of Chemical and Food Technology, Ho Chi Minh City University of Technology and Education, Ho Chi Minh City, Viet Nam

**Jin-Woo Jung,** Department of Physics and Chemistry, Daegu Gyeongbuk Institute of Science & Technology (DGIST), Daegu 42988, South Korea

**Keywords:** strong coupling, exciton-polaritons, self-hybridized optical microcavities, organic-inorganic lead halide perovskites

**
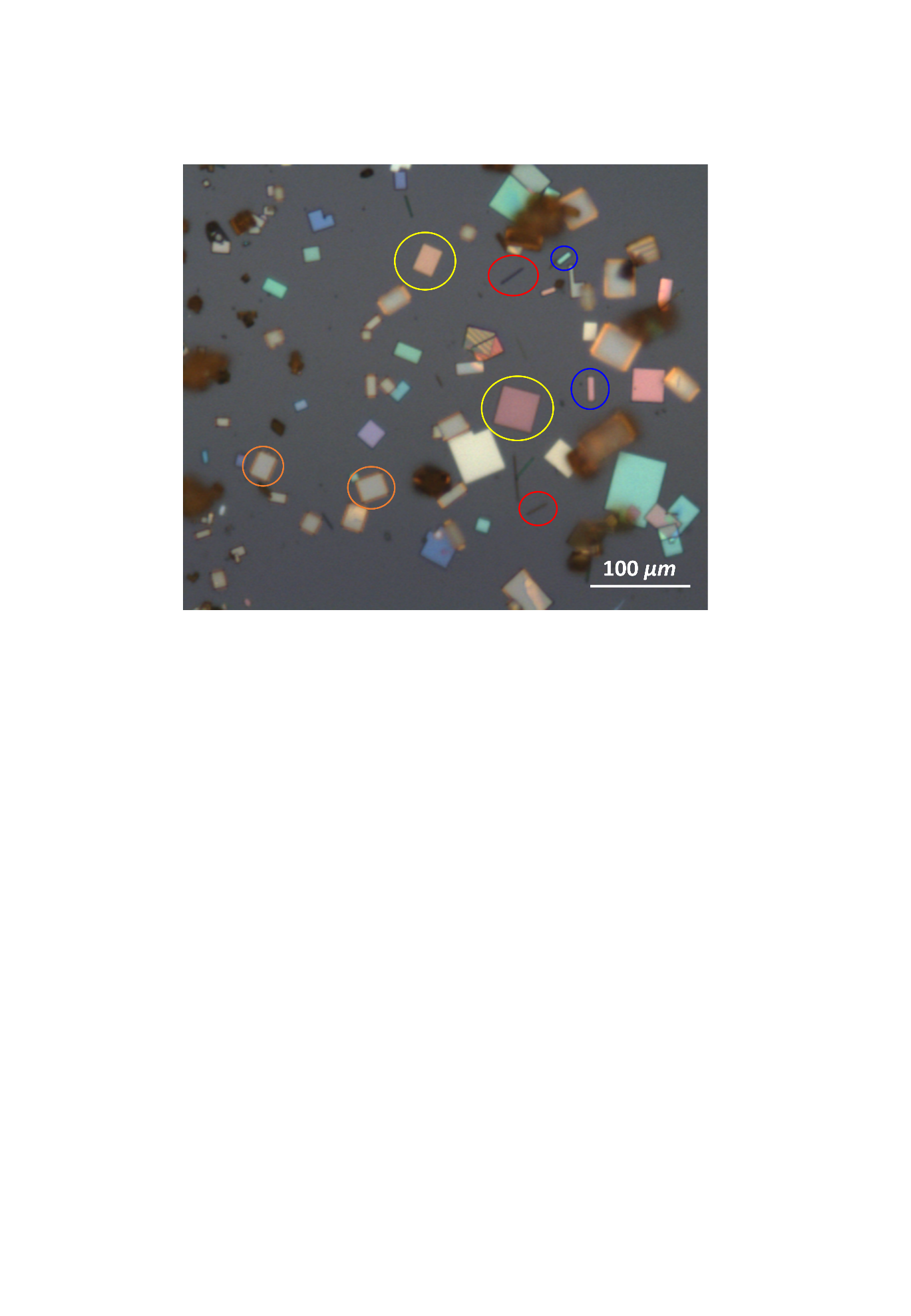
**

**Figure S1:** Bright field optical microscope image showing CH_3_NH_3_PbBr_3_ microcrystals with different morphologies such as thick (orange) / thin (yellow) MPs, MR (blue) and micro/nanowires (red), all grown via the spaced confined anti-solvent crystallization technique.


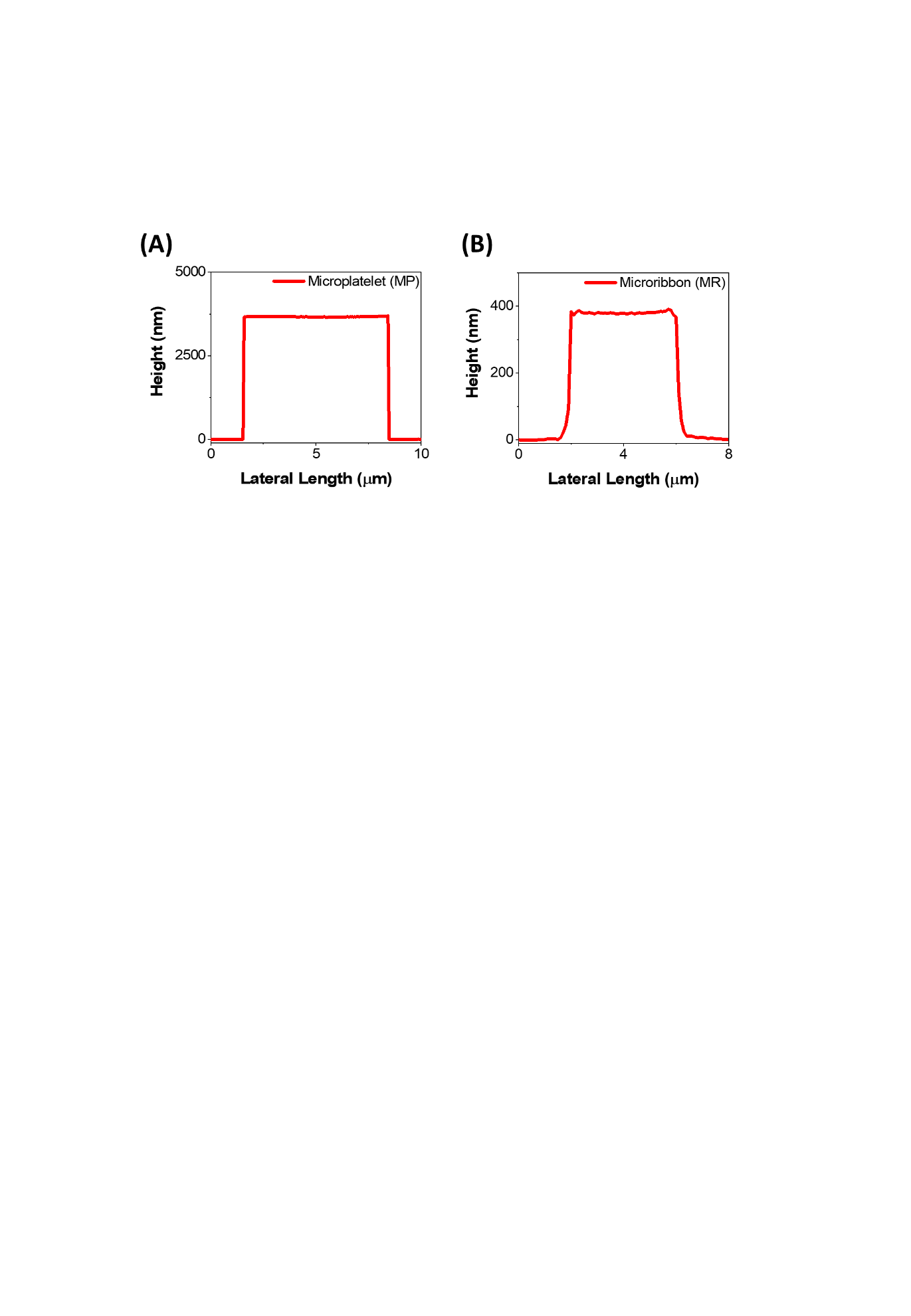


**Figure S2**: Height profiles. (A, B**)** The confocal microscope height profiles of perovskite MP and MR geometries indicate a thickness (height) of ~ 3.67 and 0.378 µm, respectively.

**Table S1:** Peak positions and corresponding FWHM of the multiple resonances observed in the PL spectra of the perovskite geometries.

| **Microplatelet (MP)** | | **Microribbon (MR)** | |
| --- | --- | --- | --- |
| Peak Position (eV) | FWHM (meV) | Peak Position (eV) | FWHM (meV) |
| 2.3063 | 38.3 | 2.3103 | 35.9 |
| 2.2857 | 18.5 | 2.2933 | 24.6 |
| 2.2614 | 22.8 | 2.2762 | 22.8 |
| 2.2270 | 26.4 | 2.2535 | 23.2 |
| 2.1835 | 28.2 | 2.2238 | 29.7 |
| 2.1316 | 35.7 | 2.1889 | 23.9 |

**Note S1: ARR spectroscopy of MP and MR**

**Figure S3(A)** presents the ARR mapping of the perovskite MP. The mapping shows a series of clear parabolic dispersions (black color) consistent with ARPL mapping of **Figure 4C** (main text). In contrast, the ARR mappings of MR geometry (**Figure S3(B)** and **(C**)) neither display the parabolic dispersions nor the interference patterns, contrary to ARPL counterpart (**Figure 4D** and **5A**: main text). This is because ARR signal/mapping stems from the absorption of white light incident along the *z*-axis (perpendicular to *x-y* plane) of the sample and therefore will only display the polariton modes coupled to cavity photons confined in the out-of-plane orientation (z-axis). In this regard, since polariton modes in MP geometry are coupled to cavity photons confined along the *z*-axis, therefore they were displayed in ARR mapping (**Figure S3(A))** accordingly. Conversely, the polariton modes in MR geometry are coupled to the cavity photons confined along the in-plane (*x*-*y*) orientation. Consequently, they were not observed in ARR mappings.

To further validates this phenomenon, we prepared a ~1.45 $\mu$m thick MR such that an additional FP cavity is formed along *z*-axis (akin to MP geometry) in conjunction with the conventional waveguide type FP cavity of the MR geometry along the *x-y* orientation. **Figure S3(D)** and **S3(E)** present ARPL mappings, wherein the bright/strong parabolic dispersions represents the lower polariton modes formed due to strong coupling between excitons and FP cavity modes confined along the *z*-axis, while the relatively weaker parabolic dispersions (**Figure S3(D)**) and the associated interference patterns (**Figure S3(E)**) corresponds to lower polariton modes formed due to strong coupling between excitons and the waveguide type FP modes confined along the in-plane (*x-y*) axis of the MR geometry. Interestingly, the ARR mapping (**Figure S3(F)**) of MR geometry only displays the polariton modes coupled to FP cavity modes confined along the *z*-axis, which is consistent with **Figure S3(A), (B)** and (**C)** as well.


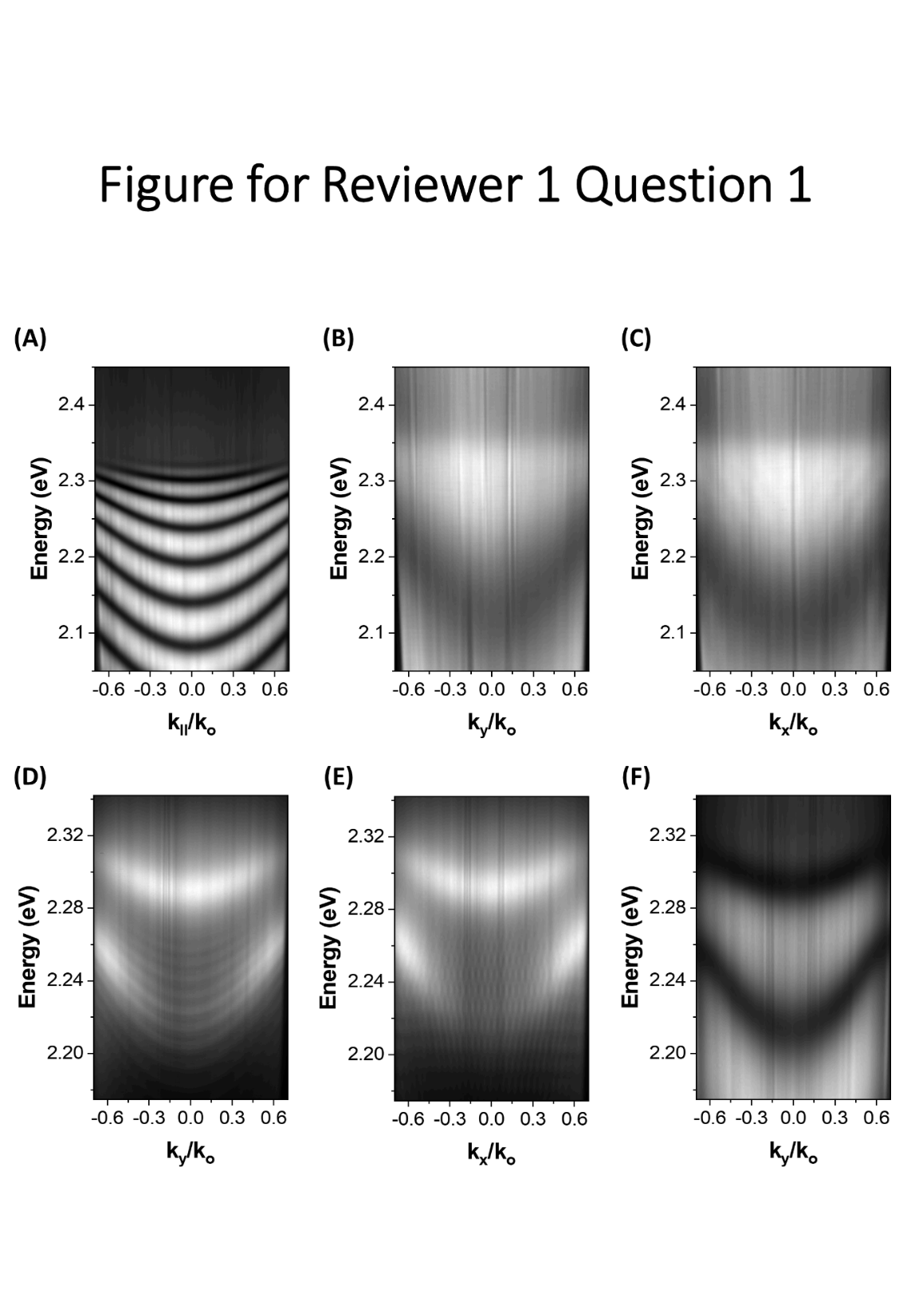


**Figure S3:** Angle-resolved reflectance (ARR) and ARPL mappings. (A) The ARR mapping of MP geometry, wherein the parabolic dispersions (color: black) represent the LPBs formed due to strong coupling between excitons and multiple FP cavity modes. (B and C) ARR mapping acquired when *y* and *x*-axis of MR is set parallel to the spectrometer slit, respectively. The ARR mappings of MR neither displays the parabolic dispersions nor the interference patterns, because ARR spectroscopy only detects the polariton modes coupled to photons confined in the out of plane orientation. Since, polaritons in MR geometry are coupled to photons confined along the in-plane direction. Resultantly, they were not observed in ARR mappings. (D and E) ARRL mapping when *y* and *x*-axis of MR is set parallel to the spectrometer slit, respectively. Owing to its relatively large thickness (*z*-dimension) compared to one reported in main text (**Figure 4D** and **5A**), an additional simple FP cavity is formed along the *z*-axis (akin to MP geometry) together with the conventional waveguide type FP cavity along the in-plane (*x-y*)-axis. Consequently, ARPL mappings display both the polariton modes associated with photons confined along the out-of-plane (z-axis) and the in-plane (*x-y*)-axis. For instance, the strong/bright parabolic dispersions in these mappings (**Figure S3(D)** and **(E)**) correspond to polariton coupled to cavity photon confined along the z-direction. While the weaker parabolic dispersions in **Figure S3(D)** and their representative interference patterns in **Figure S3(E)** represent the polariton modes coupled to cavity photons confined along the *x*-*y* axis. (F) ARR mapping of the 1.45 µm thick (*z-*axis) and ~12 µm wide (*x-*axis) MR geometry. As expected, the mapping only displays the polariton modes coupled with photons confined along the *z*-axis, consistent with **Figure S3(A)**, **(B)** and **(C)**.

**Note S2: Experimental vs TMM simulated ARR mapping of perovskite MP**

**Figure S4** presents the experimental (left) and TMM simulated (right) reflectance mapping of the MP geometry. The mappings clearly display a good agreement between the experimental and TMM simulated LPBs (black color parabolic dispersion). Note that for TMM simulation of the polariton in the MP geometry, the refractive index of the CH_3_NH_3_PbBr_3_ is derived from the dielectric function (denominator of the equation S3) modelled via the Lorentz oscillator model following the parameter mentioned in Note S6.


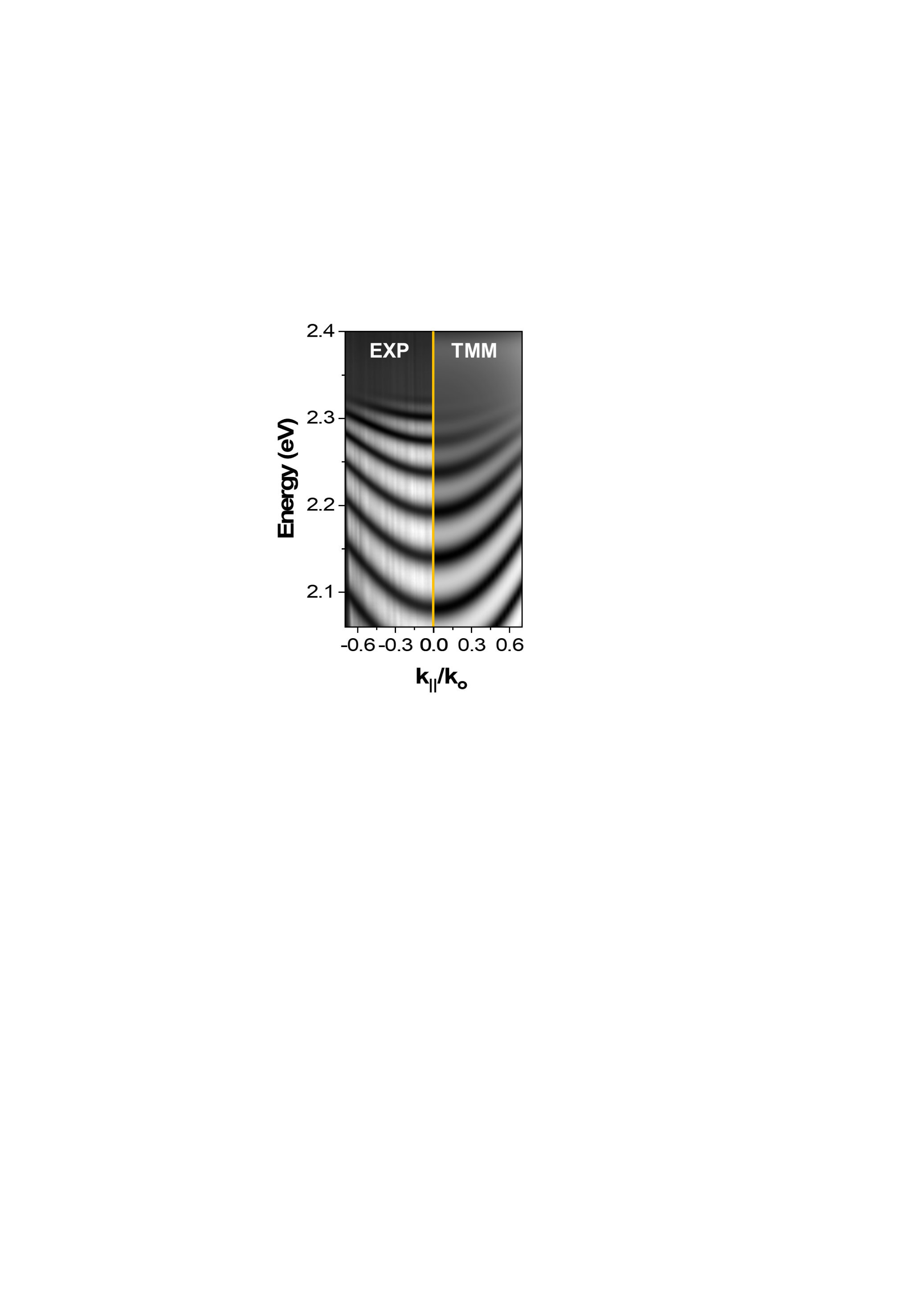


**Figure S4:** Comparison of the experimental (left) and the TMM simulated (right) ARR mapping of the MP geometry.

**Note S3:** **Calculation of the uncoupled cavity modes**

**Micro-platelet (MP)**

As shown schematically in **Figure 2C**, MP forms a simple FP cavity along the *z*-axis. The uncoupled FP cavity modes (*E_CM_*) of MP were estimated using [1]

$E_{CM}=\frac{\hbar c}{n_{c}}\frac{j\pi}{L}\left\{ 1-\left( \frac{\sin\theta}{n_{c}} \right)^{2} \right\}^{-\frac{1}{2}}$ equation (S1)

where *ħ, c, n_c_, j,* and *L* indicate the reduced Planks constant, speed of light, cavity refractive index, mode number, and the length of the FP cavity, respectively, and sin *θ* = NA (numerical aperture of microscope objective). For *n_c_* = 2.288, *L* = 3.67 µm, and *j* = (29 - 34), the calculated modes ($E_{CM}$) of the MP geometry are shown in **Figure S5(A).**

**Micro-ribbon (MR)**

As mentioned in the main text, MR forms an FP-type waveguide microcavity (similar to a slab waveguide, but with a reduced lateral dimension), wherein the guided modes travel along the lateral (*x*-*y*) dimension via total internal reflection in a zig-zag fashion forming an FP cavity along the shorter direction (*x*-axis) as shown schematically in **Figure 2D**.

In this regard, the energy of the uncoupled cavity modes ($E_{CM}$) of the MR geometry can be described analytically via the characteristic equation as [2, 3]

$k_{z1}d=2\arctan\left( k_{z2}/k_{z1} \right)+m\pi$ equation (S2)

$$k_{z1}=\sqrt{k_{0}^{2}n_{bg}^{2}-\delta^{2}}$$

$$k_{z2}=\sqrt{\delta^{2}-k_{0}^{2}}$$

$$E_{CM}=\frac{c\hbar k_{0}}{\cos\left( \arcsin\left( 1/n_{bg}\sin\theta\right) \right)}$$

where $k_{z1}$ and $k_{z2}$ are the wavevectors inside and outside of the MR cavity along the *z*-axis, respectively, while *d*, *m*, *k*_o_, and *n_bg_* represent the thickness of MR, mode order of the guided modes, wavevector in free space, and background refractive index, respectively. Moreover, *k*_o_*n*_bg_ corresponds to the wavevector of the guided mode, while *β* represents the propagation constant along the *x*-axis of MR. For MR with width (*W*), $\delta$ satisfies the FP resonance condition i.e., $\delta=\frac{N}{W}\pi$, where *N* is the integer number for each mode. Note that MR only supports the modes that satisfy both the characteristic equation (S2) for the guided modes and the FP resonance condition, simultaneously. The uncoupled cavity modes (*E_CM_*) of MR were obtained by simultaneously fitting the multiple dispersions in the ARPL mapping (**Figure 4D**) for $g$ = 0.1175 eV and *n*_bg_ = 2.288 as shown in **Figure S5(B)**.

**
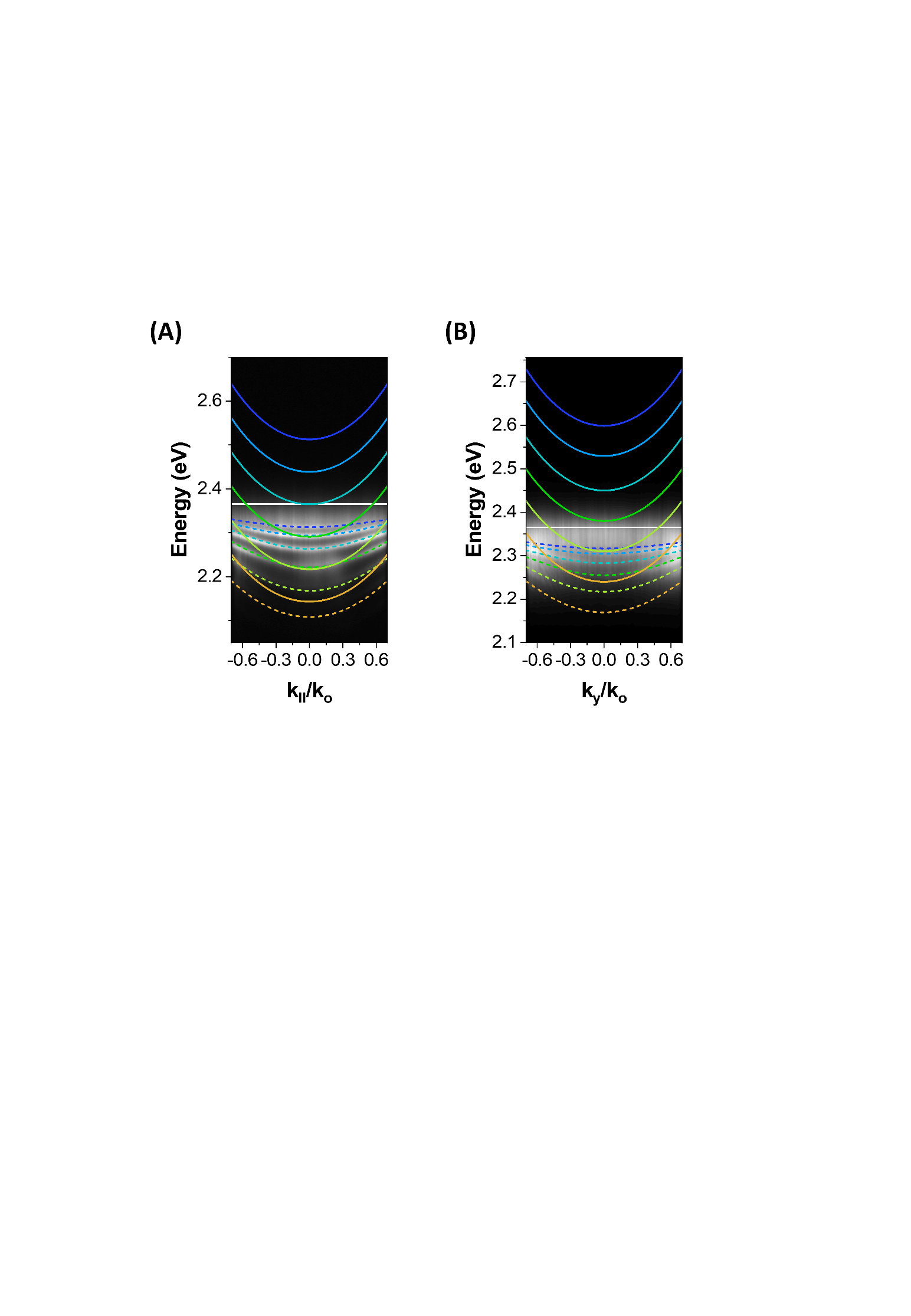
**

**Figure S5:** Dispersion of lower polariton branches and their associated uncoupled cavity modes. (A) The uncoupled cavity modes (solid lines) of the MP cavity were calculated using equation (S1). (B) The uncoupled cavity modes of the MR cavity (solid lines) were estimated by fitting LPBs (dotted traces) in the ARPL mapping via COM for $g$= 0.1175 eV and *n*_bg_ = 2.288.


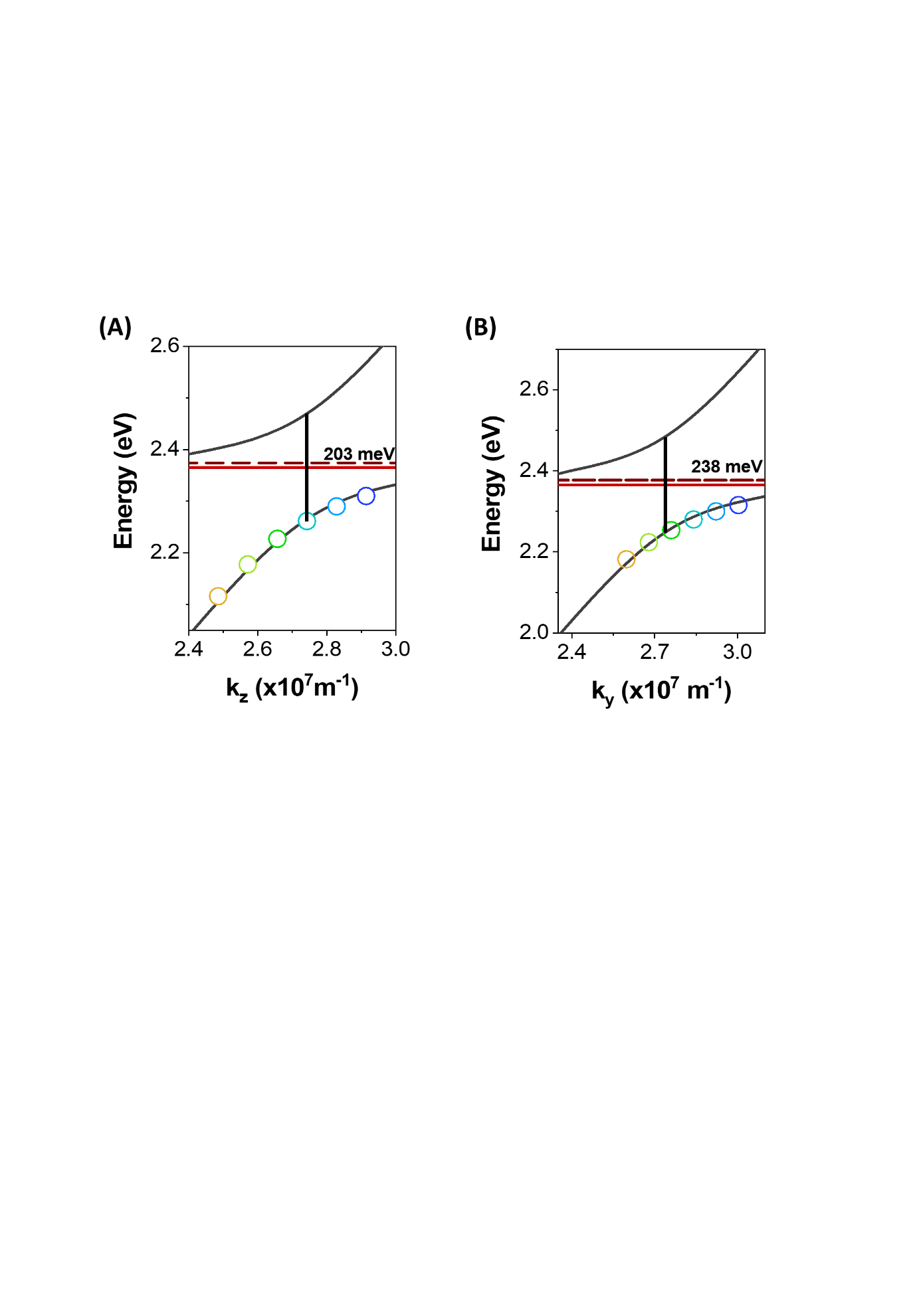


**Figure S6:** *E-k* plots. (A and B) The calculated *E-k_z_* and *E-k_y_* plots of MP and MR geometry, respectively.

**Note S4: *E-k* dispersion calculation**

The polariton dispersion or *E-k* curves for the perovskite MP and MR geometry were approximated by the two Lorentz oscillator model expressed mathematically as

$E\left( k \right)=\hbar ck\left\{ \epsilon_{b}\left( 1+\frac{E_{L}^{2}-E_{T}^{2}}{E_{T}^{2}-E^{2}-ⅈE\hbar\gamma} \right) \right\}^{-1/2}$ equation (S3)

where $\epsilon_{b}$ is the background permittivity, *E_L_* and *E_T_* are longitudinal and transverse resonance energy, respectively while *γ* is the exciton damping or decay constant. In addition, *E* represent the center value of the multiple resonance peaks in the steady state PL spectrums while the wavevector (*k*) = *k_o_* + nπ/*L*, where *k_o_* is the fitting parameter indicating the parallel shift of *E* in the momentum axis and *L* is the thickness/width of MP/MR.

Following equation S3, the polariton dispersion (solid: black line) is best calculated for $\epsilon_{b}$ = 5.23, [4] *E_L_* = 2.374 eV, *E_T_* = 2.365 eV, γ = 30 meV [5] and *L* = 3.67 µm in case of/for MP and $\epsilon_{b}$ = 5.23, *E_L_* = 2.377 eV, *E_T_* = 2.365 eV, γ = 30 meV and *L* = 3.78 µm for MR as shown in **Figure S6(A)** and **(B),** respectively. The scattered data points in these plots correspond to the peak positions of the multiple resonances in the experimentally measured PL spectrums shown in **Figure 4(A)** and **(B)** for MP and MR, respectively. Note that initial positions of these data points were roughly estimated by *k_y_* = nπ/*L*, and then fitted to the calculated polariton dispersion (solid: black line) via a parallel shift (*k*_o_) along the momentum axis. Clearly, the data points match well with LPB of the calculated polariton dispersion (solid: black line), thereby confirming that the multiple resonances observed in **Figure 4(A)** and **(B)** are indeed the lower polariton modes. Furthermore, **Figure S6(A)** and **(B)** demonstrate a clear anti-crossing at the exciton resonance with a rabi splitting energies (*ħΩ*) ~203 and 238 meV for MP and MR, evaluated from the minimum vertical distance between the upper and lower polariton branches. Interestingly, these values are consistent with their respective *ħΩ* values acquired from COM fitting i.e., 205 and 235 meV.

**
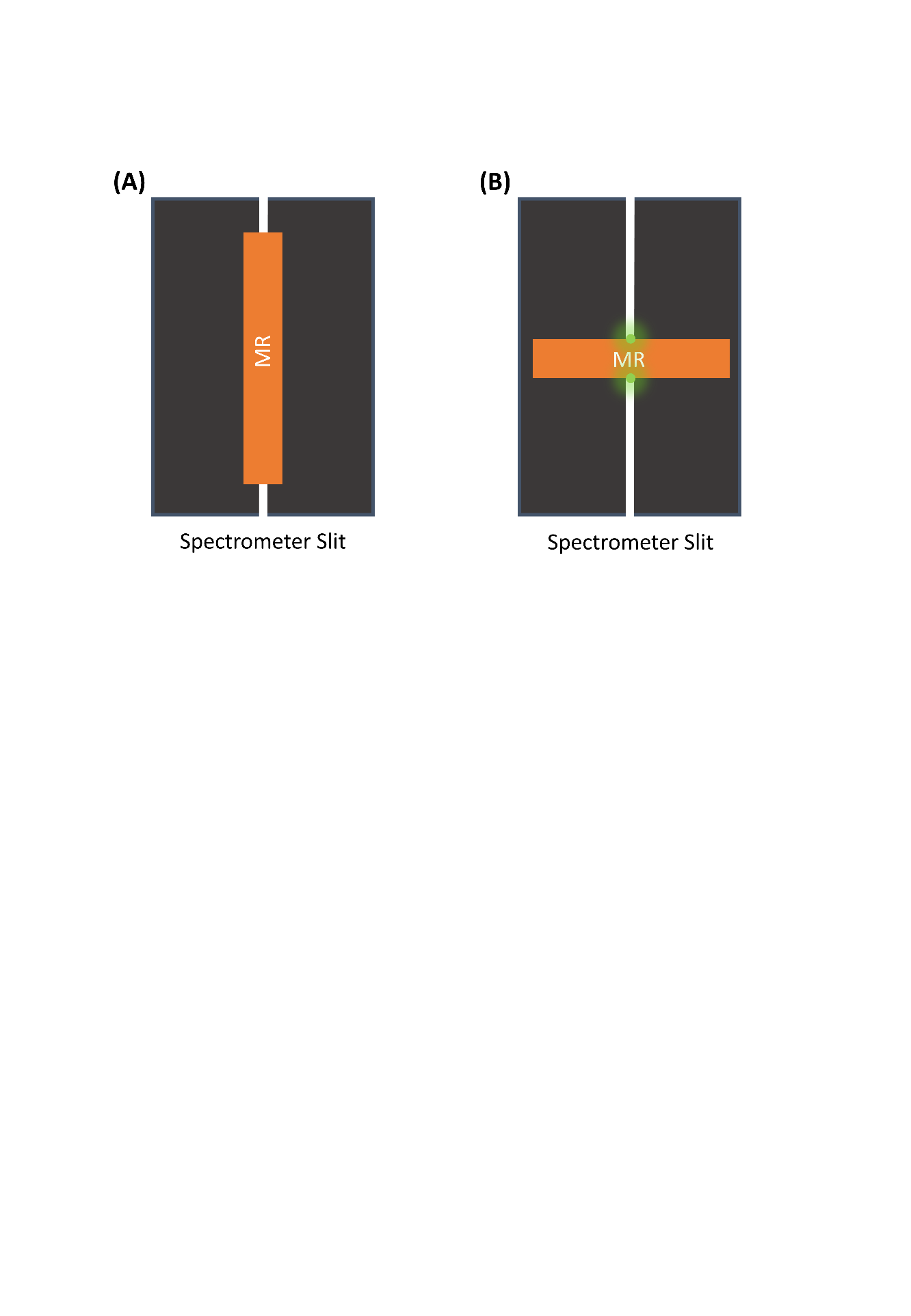
**

**Figure S7:** Schematic representation of MR orientation with respect to the spectrometer slit. (A) The long side (*y*-axis) of the MR is set parallel to the spectrometer slit. (B) The shorter side (*x*-axis) of the MR is set parallel to the spectrometer slit.

**Note S5:** **Equations for even and odd modes in Young’s double slit experiment**

The interference spectra acquired from the ARPL mapping of the MR geometry can be described by the following equations [2].

$I\left( \theta\right)=I_{0}{cos}^{2} \left( \pi\frac{L\sin\theta}{\lambda}+\frac{\pi}{2} \right)$, for odd modes (S4)

$I\left( \theta\right)=I_{0}{cos}^{2} \left( \pi\frac{L\sin\theta}{\lambda} \right)$, for even modes (S5)

where *I* is the intensity of the interference pattern with *I_0_* being the overall scaling factor and *L* = 3.870 µm is the distance between the point sources (width of MR), respectively.

**Note S6:** **Rabi splitting vs cavity quality factor**

To examine the evolution of coupling strength and quality factor relative to microcavity length i.e. (*z*-axis for MP and *x*-axis for MR). We performed ARPL and steady-state PL spectroscopy for both MPs and MRs geometries with varying *z* and *x*-axis dimensions as shown in **Figure S8** and **S9**, respectively. The experimentally measured ARPL mappings were fitted via COM to estimate the coupling strength (*g*) and rabi-splitting energies (*ħΩ = 2g*) while their corresponding PL spectrums were fitted by multi-Lorentz function to calculate the cavity quality factor (*Q*) following the equation *Q* = *λ*/Δ*λ*, where *γ* refers to the center wavelength while Δ*λ* corresponds to FWHM of the peak.

**Figure S10(A)** and (**B)** present “*ħΩ”* vs “*Q*“ plots acquired from eight different MPs and MRs with varying *z*-axis (thickness) and *x*-axis (widths) dimensions, respectively. The lateral (*x-y*) dimensions of MPs were in the range (50 x 60) ± 30 µm^2^ while the *y*-axis of the MRs is ~20 ± 10 µm^2^ and *z*-axis is ~0.200 ± 0.150 µm^2^. Clearly, *ħΩ* for both MPs and MRs increase with the increase in *Q.* For instance, *ħΩ* for MP increases from 217 to 300 meV as *Q* goes 82.4 to 240. Similarly, *ħΩ* for MR increases from 232 to 310 meV as *Q* goes from 110 to 221, signifying that coupling strength between excitons and cavity photons is directly proportional to cavity quality factor.


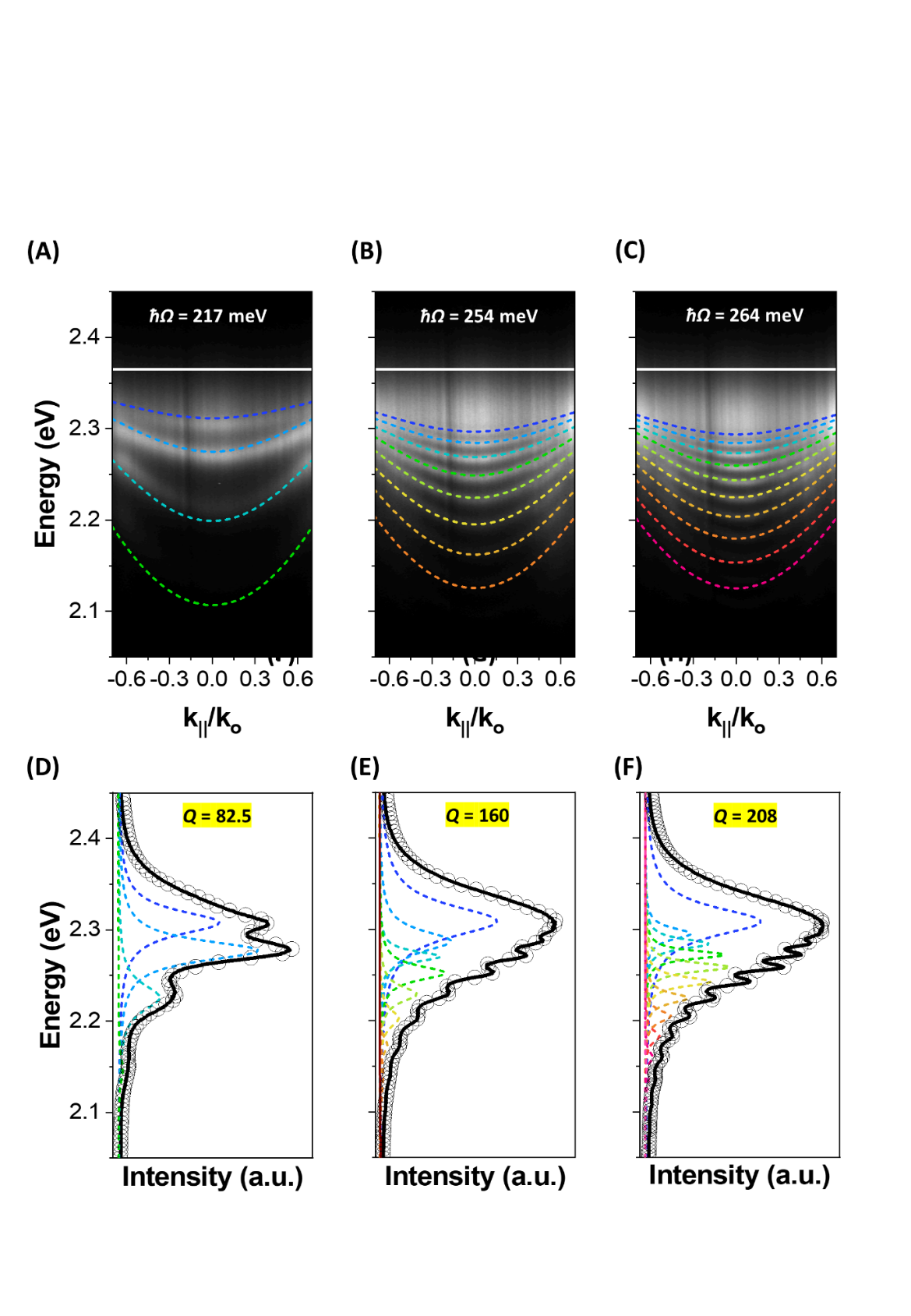


**Figure S8:** ARPL mappings and steady state PL spectra of MPs with varying thicknesses. (A, B and C) COM fitted ARPL mappings of 2.14, 5.56 and 7.15 µm thick MPs. (D, E and F) The respective steady state PL spectrums indicate quality factor of ~82.5, 160 and 208, respectively.


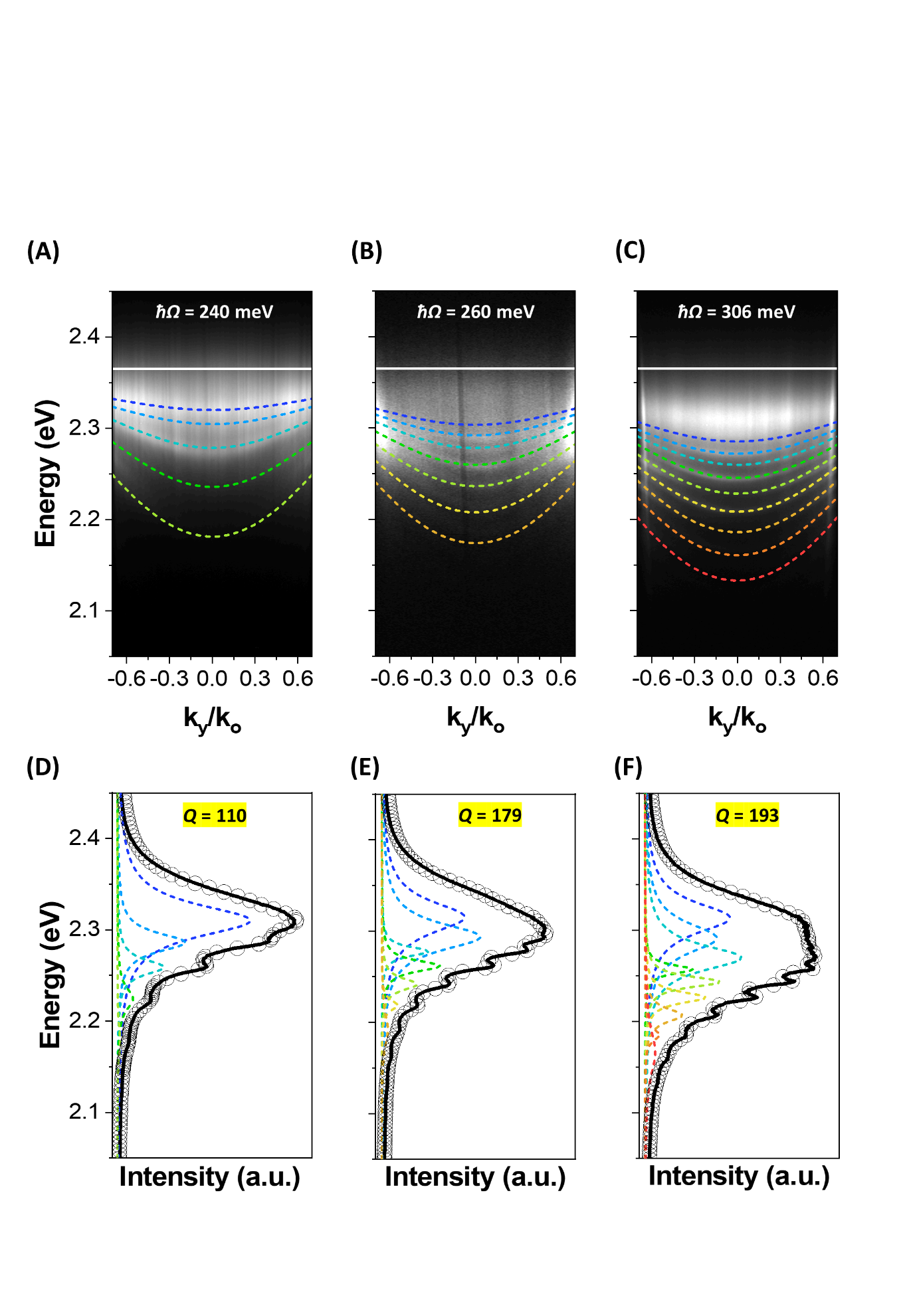


**Figure S9:** ARPL mappings and steady state PL spectra of MRs with varying widths. (A, B and C) COM fitted ARPL mappings of 2.77, 5.15 and 6.55 µm wide MRs. (D, E and F) The corresponding steady state PL spectrums demonstrate a quality factor of ~110, 179 and 193, respectively.


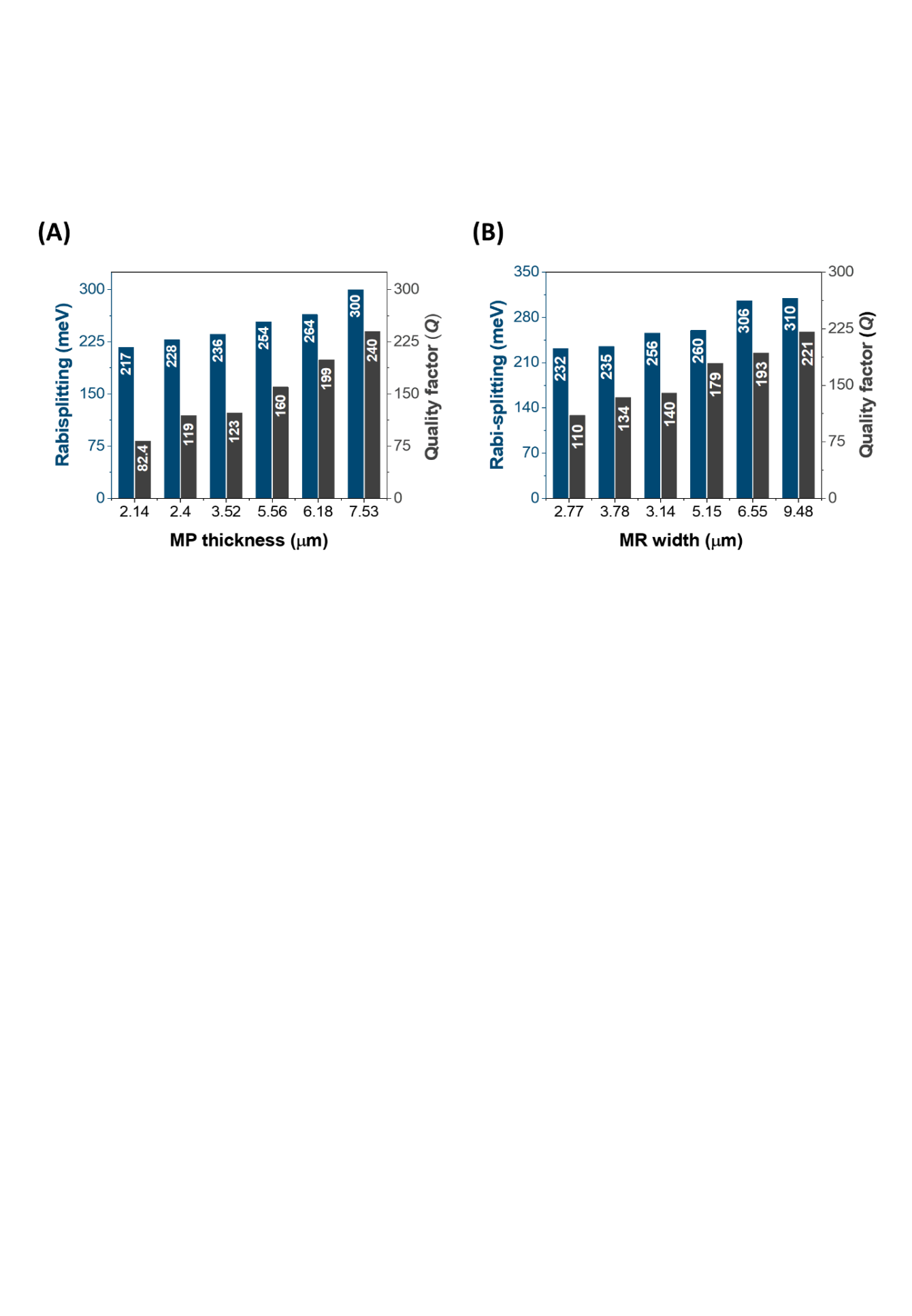


**Figure S10:** *ħΩ* vs *Q* plots. (A and B) These plots demonstrate the relationship between *ħΩ* and *Q* of different MPs and MRs with varying thicknesses and widths, respectively. Clearly, *ħΩ* increases with increase in *Q* for both MP and MR.

**References**

1. Q. Han, J. Wang, J. Lu, et al., "Transition between exciton-polariton and coherent photonic lasing in all-inorganic perovskite microcuboid," *ACS Photonics*, vol. 7, pp. 454-462, 2020. https://doi.org/10.1021/acsphotonics.9b01413.
2. L. Sun, M. L. Ren, W. Liu, and R. Agarwal, "Resolving Parity and Order of Fabry–Pérot Modes in Semiconductor Nanostructure Waveguides and Lasers: Young’s Interference Experiment Revisited," *Nano Lett*., vol. 14, pp. 6564-71, 2014. https://doi.org/10.1021/nl503176w.
3. J. Tang, J. Zhang, Y. Lv, et al., "Room temperature exciton–polariton Bose–Einstein condensation in organic single-crystal microribbon cavities," *Nat. Commun.,* vol. 12, pp. 1-8, 2021. https://doi.org/10.1038/s41467-021-23524-y.
4. S. Brittman, and E. C. Garnett, " Measuring n and k at the Microscale in Single Crystals of CH_3_NH_3_PbBr_3_ Perovskite," *J. Phys. Chem. C,* vol. 120, pp. 616-620, 2016. https://doi.org/10.1021/acs.jpcc.5b11075.
5. F. Mariano, A. Cretì, L. Carbone, et al., “The enhancement of excitonic emission crossing saha equilibrium in trap passivated CH_3_NH_3_PbBr_3_ perovskite,” *Commun. Phys.,* vol. 3, p. 41, 2020. https://doi.org/10.1038/s42005-020-0309-3.
